# LAP1 Interactome Profiling Provides New Insights into LAP1’s Physiological Functions

**DOI:** 10.3390/ijms252413235

**Published:** 2024-12-10

**Authors:** Cátia D. Pereira, Guadalupe Espadas, Filipa Martins, Anne T. Bertrand, Laurent Servais, Eduard Sabidó, Philippe Chevalier, Odete A. B. da Cruz e Silva, Sandra Rebelo

**Affiliations:** 1 Institute of Biomedicine (iBiMED), Department of Medical Sciences, University of Aveiro, 3810-193 Aveiro, Portugal; daniela.pereira@ua.pt (C.D.P.); samartins@ua.pt (F.M.); odetecs@ua.pt (O.A.B.d.C.e.S.); 2 Center for Genomics Regulation, The Barcelona Institute of Science and Technology (BIST), 08003 Barcelona, Spain; guadalupe.espadas@crg.eu (G.E.); eduard.sabido@crg.eu (E.S.); 3 Department of Medicine and Life Sciences, Universitat Pompeu Fabra, 08003 Barcelona, Spain; 4 Centre de Recherche en Myologie, Institut de Myologie, Medicine Faculty—Sorbonne Université, Inserm, 75013 Paris, France; a.bertrand@institut-myologie.org; 5 MDUK Oxford Neuromuscular Center, Department of Paediatrics, University of Oxford and NIHR Oxford Biomedical Research Center, Oxford OX3 9DU, UK; laurent.servais@paediatrics.ox.ac.uk; 6 Neuromuscular Center, Division of Paediatrics, University Hospital of Liège and University of Liège, 4000 Liège, Belgium; 7 Institut NeuroMyoGène (INMG), Université Claude Bernard Lyon 1, 69266 Lyon, France; philippe.chevalier@chu-lyon.fr; 8 Hospices Civils de Lyon, 69677 Lyon, France

**Keywords:** proteomics, protein–protein interactions, bioinformatics, heterochromatin assembly, DNA repair, proteostasis

## Abstract

The nuclear envelope (NE), a protective membrane bordering the nucleus, is composed of highly specialized proteins that are indispensable for normal cellular activity. Lamina-associated polypeptide 1 (LAP1) is a NE protein whose functions are just beginning to be unveiled. The fact that mutations causing LAP1 deficiency are extremely rare and pathogenic is indicative of its paramount importance to preserving human health, anticipating that LAP1 might have a multifaceted role in the cell. Mapping the LAP1 protein interactome is, thus, imperative to achieve an integrated view of its potential biological properties. To this end, we employed in silico- and mass spectrometry-based approaches to identify candidate LAP1-interacting proteins, whose functional attributes were subsequently characterized using bioinformatics tools. Our results reveal the complex and multifunctional network of protein–protein interactions associated to LAP1, evidencing a strong interconnection between LAP1 and cellular processes as diverse as chromatin and cytoskeleton organization, DNA repair, RNA processing and translation, as well as protein biogenesis and turnover, among others. Novel interactions between LAP1 and DNA repair proteins were additionally validated, strengthening the previously proposed involvement of LAP1 in the maintenance of genomic stability. Overall, this study reaffirms the biological relevance of LAP1 and the need to deepen our knowledge about this NE protein, providing new insights about its potential functional partners that will help guiding future research towards a mechanistic understanding of LAP1’s functioning.

## 1. Introduction

Lamina-associated polypeptide 1 (LAP1) is an ill-defined protein of the nuclear envelope (NE), a specialized double-membrane system sculpted from the endoplasmic reticulum (ER) that encloses the nuclear genome. Being a distinctive feature of eukaryotic cells, the NE plays a key role in the control of biological events in the nucleus (e.g., DNA transcription, replication and repair) and as a mediator of nucleocytoplasmic crosstalk. Whereas the outer nuclear membrane (ONM) of the NE is physically interconnected and shares some structural constituents with the main ER, the inner nuclear membrane (INM), due to its affinity for the nuclear lamina and chromatin, preserves a specific proteome that endows the NE with the ability to carry out unique functions in the cell [1]. As an INM-localized lamina-/chromatin-binding protein [2], LAP1 certainly contributes to this important functional specialization of the NE.

Encoded by the human *TOR1AIP1* gene, LAP1 is a type II transmembrane protein that displays a ubiquitous expression [3] and presents as different isoforms in mammalian cells/tissues. LAP1A, LAP1B and LAP1C isoforms arising from alternative messenger RNA (mRNA) splicing were initially described in rat [4] and mouse [5] species. In humans, only LAP1B [6] and LAP1C isoforms have been identified, whose production is regulated by alternative transcription initiation [3]. Structurally, mammalian LAP1 isoforms are predicted to have a similar membrane topology. Being integrated into the INM through a single membrane-spanning domain, LAP1 is additionally composed of two other segments lying at opposing sides of the INM [5,6] that mediate its binding to cellular components: (i) a N-terminal domain facing the nucleoplasm [5,6], via which LAP1 associates with chromatin [2], lamins A/C and B1 [2,7], protein phosphatase 1 (PP1) [8], emerin [9] and, presumably, telomeric repeat-binding factor 2 (TRF2) [10]; and (ii) a C-terminal domain that faces the NE lumen separating the two nuclear membranes [5,6], known to be involved in the formation of LAP1:torsinA and LAP1:torsinB complexes [11,12,13].

Evidence has accumulated throughout the years pointing to the multifunctional nature of LAP1. For instance, previous reports have suggested its involvement not only in tissue-specific processes, namely myogenic differentiation [14], spermatid maturation [15] and neuromuscular transmission [16], but also in more general cellular activities, such as mitosis [17,18]. Many valuable insights into the physiological properties of LAP1 have emerged from studies that investigated its interaction with other proteins. One of the most prominent examples concerns the LAP1-binding partner torsinA, an ER/NE lumen-resident ATPase that requires LAP1 as a cofactor for the activation of its ATP hydrolysis activity at the NE [12,13,19]. Further examination of the biological relevance of their interplay revealed that LAP1 cooperatively acts with torsinA to control the coordinated positioning of the nucleus and centrosome during cell migration [20], as well as to regulate hepatic lipid metabolism by modulating the secretion of very-low-density lipoprotein (VLDL) particles [21]. Moreover, the functional characterization of LAP1 interactors using bioinformatics tools, as performed by Serrano and colleagues in 2016 [22], is another good example of how the analysis of LAP1-binding partners can shed light on its functioning. In this in silico study, it was uncovered that the LAP1 interactome was enriched with proteins involved in the regulation of response to DNA damage stimulus, as it was the case of TRF2 [22]. These data prompted new investigations by our group, wherein we found that the LAP1:TRF2 interaction is induced under genotoxic conditions and these proteins spatially overlap with DNA damage response (DDR) factors in human cells, indicating that LAP1 could have an uncharacterized function in DDR/DNA repair [10].

Deciphering LAP1’s biological roles is of great clinical importance due to the association of loss-of-function *TOR1AIP1* mutations with the development of severe human diseases [16,23,24,25,26,27,28,29,30,31]. Besides resorting to the identification of its interaction partners to clarify how LAP1 operates in a physiological scenario, another means to address this question is by unraveling the pathological consequences of its dysfunction. Recently, our group performed a quantitative proteome analysis of patient-derived skin fibroblasts carrying the LAP1 E482A missense mutation [32], which has previously been linked to a lethal phenotype consisting of dystonia, dilated cardiomyopathy and cerebellar atrophy [24]. Using LAP1 E482A fibroblasts as a human LAP1 knockdown cell model, as they exhibit reduced protein levels of LAP1B and LAP1C isoforms [24], we identified several differentially expressed proteins in these patient’s cells relatively to control skin fibroblasts and discovered that LAP1 depletion potentially causes perturbations in DNA repair, translation, protein folding/degradation and oxidative stress response [32]. Notably, considering that LAP1 has already been proposed to participate in DDR/DNA repair [10], these results raised the interesting possibility that it might also be implicated in the remaining biological activities deregulated in LAP1 E482A fibroblasts.

In this work, we characterized the LAP1 interactome network to gain deeper knowledge on the functional features of this INM protein. In addition to conducting a bioinformatic search for known interactions, an experimental approach combining co-immunoprecipitation and liquid chromatography with tandem mass spectrometry (LC–MS/MS) techniques was used to identify novel LAP1-binding proteins in human cells. In silico functional enrichment analyses of Gene Ontology (GO) categories and signaling pathways associated to the interactors were carried out to determine the most relevant physiological processes wherein LAP1 might be an active player. To help decoding its potential mechanisms of action, we also investigated functional and pathological relationships between the LAP1-interacting partners reported here and the differentially expressed proteins previously identified in LAP1 E482A fibroblasts by our group [32], through an integrative bioinformatic analysis of protein–protein interactions (PPIs) and gene–disease associations.

## 2. Results

**Figure 1:**
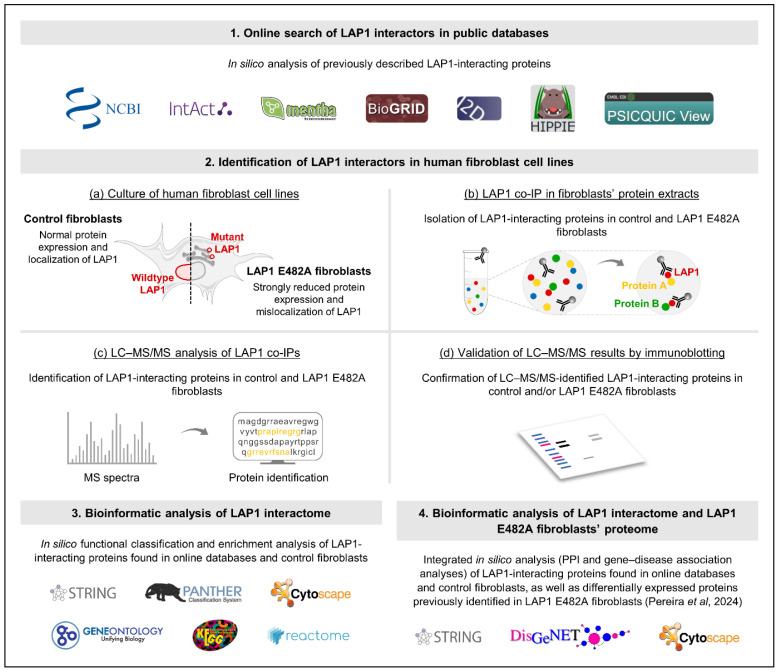
Schematic representation of the experimental design of this study [32]. IP, immunoprecipitation; LAP1, lamina-associated polypeptide 1; LC–MS/MS, liquid chromatography coupled to tandem mass spectrometry; MS, mass spectrometry; PPI, protein–protein interaction.

Herein, we set out to identify the LAP1 protein interactome using bioinformatics- and proteomics-based approaches, as well as to characterize the functional properties of LAP1-interacting proteins in order to shed light into the biological roles of LAP1 and its importance in human physiology. In addition, we undertook a comprehensive analysis to uncover relevant physiological and pathological associations between these LAP1 interactors and the deregulated proteins found in patient-derived LAP1-deficient fibroblasts bearing the disease-causing LAP1 E482A mutation, the latter of which were recently reported in a study from our group [32]. The experimental design of the present work is summarized in Figure 1.

### 2.1. Identification and Functional Characterization of LAP1 Protein Interactome

#### 2.1.1. Identification of LAP1-Interacting Proteins Through Online Search of Public Databases

**Figure 2:**
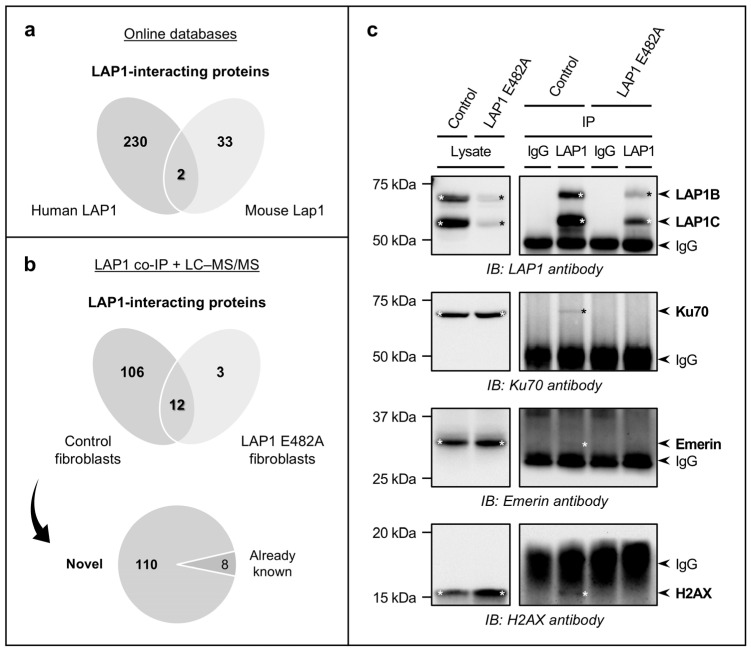
Identification of LAP1-interacting proteins. (**a**) Summarized results of the in silico analysis leading to the identification of previously known interacting proteins of human LAP1 and mouse Lap1, retrieved from online databases. (**b**) Summarized results of the LC–MS/MS analysis leading to the identification of proteins that were significantly co-immunoprecipitated with LAP1 in control and LAP1 E482A fibroblast cell lines. The number of novel LAP1-interacting proteins uncovered in control fibroblasts is also indicated. (**c**) LAP1 co-immunoprecipitation assay coupled to immunoblotting analysis validating several LC–MS/MS-identified proteins as LAP1 interactors in control fibroblasts. Whole cell lysates obtained from control and LAP1 E482A fibroblast cell lines were immunoprecipitated using a LAP1-specific antibody bound to Dynabeads Protein G. The respective negative controls were performed by incubating fibroblasts’ total protein extracts with Dynabeads Protein G-conjugated rabbit IgG. The presence of LAP1 and/or its binding partners Ku70, emerin and H2AX in the immunoprecipitates (right), as well as in the initial whole cell lysates (left), of control and LAP1 E482A fibroblasts was analyzed by immunoblotting using specific antibodies against the human target proteins; asterisks indicate visible LAP1B, LAP1C, Ku70, emerin and H2AX protein bands. IB, immunoblotting; IgG, immunoglobulin G; IP, immunoprecipitation; LAP1, lamina-associated polypeptide 1; LC–MS/MS, liquid chromatography coupled to tandem mass spectrometry.

A bioinformatic analysis was initially carried out with the objective of identifying previously known LAP1-binding partners. As explained in detail in the Materials and Methods section (see Section 4.6.1), we accessed several online public databases to collect data on experimentally tested PPIs (e.g., via yeast two-hybrid screening and/or mass spectrometry combined with affinity capture or proximity labeling techniques) between human LAP1 and other human proteins. Moreover, since the LAP1 protein sequence is conserved among human and mouse species, the human proteins with proven binding affinity for mouse Lap1 were also considered in the analysis. This in silico approach led to the identification of 232 human protein interactors of human LAP1 (Figure 2a; Supplementary Table S1) and 35 human protein interactors of mouse Lap1 (Figure 2a; Supplementary Table S2), which, upon excluding duplicated ones, resulted in a dataset of 265 different LAP1-interacting proteins retrieved from online databases.

#### 2.1.2. Identification and Validation of LAP1-Interacting Proteins in Human Fibroblast Cell Lines Through LAP1 Co-Immunoprecipitation Coupled to LC–MS/MS or Immunoblotting Analysis

Next, we endeavored to discover novel LAP1 interactors by employing a quantitative mass spectrometry-based methodology, wherein human skin fibroblasts were used as a cell model. In brief, a co-immunoprecipitation assay was firstly performed to isolate the endogenous interaction partners of LAP1 in control fibroblasts’ whole cell lysates using a LAP1-specific antibody. As an experimental control, total protein extracts were incubated with rabbit immunoglobulin G (IgG). The immunoprecipitated proteins were subjected to LC–MS/MS analysis to determine the identity of LAP1 interactors, upon which the dataset of identified proteins was filtered to select the ones that were significantly enriched in LAP1 co-immunoprecipitates versus control IgG samples, as detailed in the Materials and Methods section (see Section 4.6.1). Besides the characterization of physiological LAP1 interactions in control fibroblasts, we additionally analyzed the proteins that interact with mutant LAP1 in patient-derived LAP1 E482A fibroblasts to unveil alterations in the LAP1 interactome of these diseased cells. Overall, the proteomics experiments culminated in the identification of 118 proteins as relevant LAP1 interactors in control fibroblasts (Figure 2b; Supplementary Table S3), whereas only 15 LAP1-binding partners were found in LAP1 E482A fibroblasts (Figure 2b; Supplementary Table S4), with 12 proteins being detected in both cell lines (Figure 2b; Supplementary Table S4). Of note, studying the LAP1 interactome of control fibroblasts allowed confirming the binding affinity of LAP1 for eight previously described interactors, such as ribophorins 1 and 2, two components of the oligosaccharyl transferase (OST) complex; the protein phosphatase PP1 catalytic subunit α (PP1α); and heterogeneous nuclear ribonucleoprotein M (hnRNP M), an RNA-binding protein (Figure 2b; Supplementary Table S3). More importantly, it also permitted the discovery of 110 new LAP1-interacting proteins (i.e., proteins that were not retrieved from online databases in the in silico analysis described in Section 2.1.1), including multiple histones and ribosomal subunits (Figure 2b; Supplementary Table S3). With the purpose of validating these proteomics data, we selected a subset of proteins identified by LC–MS/MS and then verified their presence in LAP1 co-immunoprecipitation samples obtained from the two fibroblast cell lines using immunoblotting as an alternative detection method. The results showed that the NE protein emerin—a well-known LAP1 interactor—as well as the DNA repair factor Ku70 and the histone variant H2AX—two novel LAP1-binding proteins—were co-immunoprecipitated with LAP1 in control fibroblasts but not in LAP1 E482A fibroblasts (Figure 2c), which is in accordance with the LC–MS/MS findings.

#### 2.1.3. Global Functional Characterization of LAP1-Interacting Proteins Through In Silico Analysis

**Figure 3:**
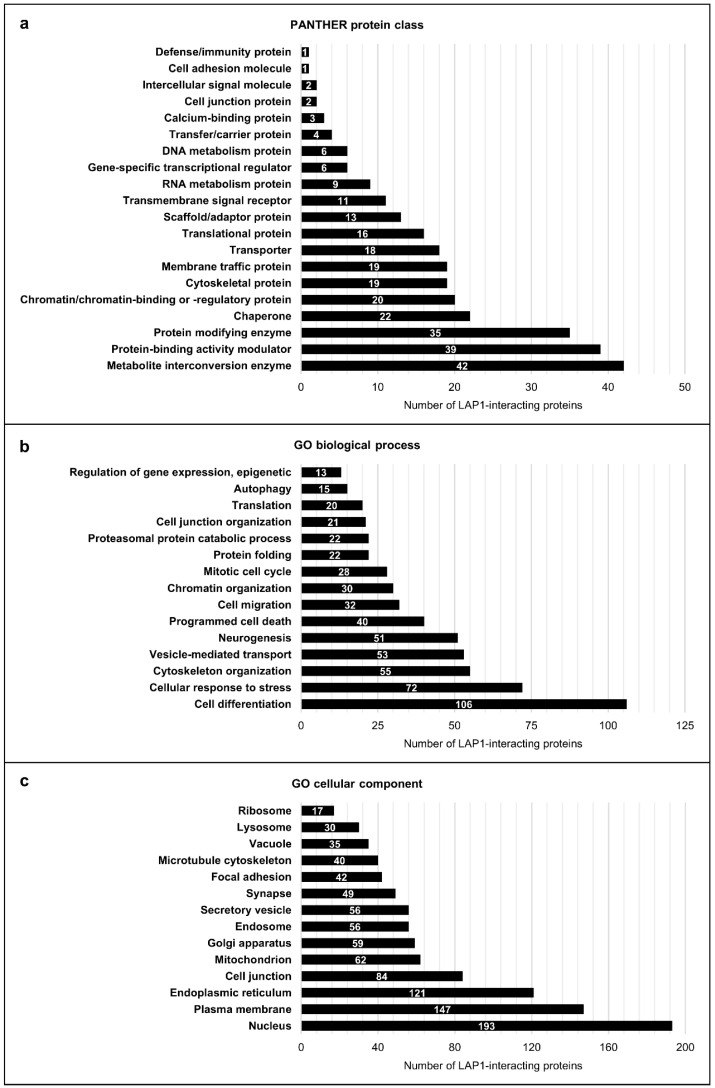
Functional characterization of LAP1-interacting proteins. (**a**) Functional classification based on PANTHER protein class categories. From a total of 375 proteins under analysis, 288 protein class hits distributed across 20 main categories were retrieved using the PANTHER online resource [33]. (**b**,**c**) Functional enrichment analysis of (**b**) GO biological process and (**c**) GO cellular component categories. Some enriched GO terms (summarizing the broad spectrum of over-represented annotations) retrieved from the STRING database [34] are shown. DNA, deoxyribonucleic acid; GO, Gene Ontology; LAP1, lamina-associated polypeptide 1; PANTHER, Protein ANalysis THrough Evolutionary Relationships; RNA, ribonucleic acid.

After identifying the LAP1 protein interactome using both in silico and experimental strategies, the data collected from online databases (Supplementary Tables S1 and S2) were combined with the LC–MS/MS data obtained from the analysis of control fibroblasts (Supplementary Table S3) to achieve the final working list of LAP1 interactors, comprising a total of 375 human proteins. To have a deeper understanding on which cellular activities might be regulated by LAP1 via association with its binding partners, we utilized bioinformatics tools to perform a global functional characterization involving all LAP1-interacting proteins (Figure 3; Supplementary Tables S5 and S6). In a first analysis, these were classified based on the protein classes that they integrate by employing the Protein ANalysis THrough Evolutionary Relationships (PANTHER) online resource [33], which retrieved protein class annotations organized into 20 main categories for 288 mapped proteins (Figure 3a; Supplementary Table S5). According to the results, many LAP1 interactors belong to the protein classes of metabolite interconversion enzymes (e.g., oxidoreductases and glycosyltransferases), protein-binding activity modulators (e.g., G proteins) and protein-modifying enzymes (e.g., proteases, ubiquitin–protein ligases, protein kinases and phosphatases) (Figure 3a; Supplementary Table S5). Chaperones, chromatin/chromatin-binding or -regulatory proteins, cytoskeletal proteins (e.g., microtubule/microtubule-binding and actin-binding proteins), membrane traffic proteins, transporters and translational proteins (e.g., ribosomal proteins and translation factors) were also among the most represented protein class categories attributed to the interaction partners of LAP1 (Figure 3a; Supplementary Table S5). In addition, a smaller number of these proteins were grouped, for example, in the protein classes of transmembrane signal receptors, RNA metabolism proteins, gene-specific transcriptional regulators (e.g., DNA-binding transcription factors) and DNA metabolism proteins (Figure 3a; Supplementary Table S5).

In a second bioinformatic analysis using the STRING database [34], the LAP1-binding proteins were further characterized by examining the most relevant annotations of GO biological process, GO molecular function, GO cellular component, Kyoto Encyclopedia of Genes and Genomes (KEGG) pathways and Reactome pathways that are assigned to them (Figure 3b,c; Supplementary Table S6). Essentially, a significant enrichment was observed for innumerous GO biological process-related terms, namely cell differentiation, cellular response to stress, cytoskeleton organization, vesicle-mediated transport, programmed cell death, chromatin organization, mitotic cell cycle, protein folding, proteasomal protein catabolic process, translation, autophagy and epigenetic regulation of gene expression (Figure 3b; Supplementary Table S6), highlighting the great diversity of physiological roles fulfilled by LAP1 interactors. In line with this, the over-represented terms of GO cellular component evidenced that the LAP1 interactome dataset is enriched with proteins that reside and/or operate at distinct subcellular locations, including the nucleus, plasma membrane, ER, cell junctions, mitochondria, Golgi apparatus, endosomes, secretory vesicles, microtubule cytoskeleton, lysosomes and ribosomes, among other intra/extracellular compartments (Figure 3c; Supplementary Table S6). Of interest, the enrichment analysis of KEGG and Reactome pathways annotations also revealed that LAP1 might intervene in several signal transduction cascades through its ability to bind proteins that take action, for instance, in insulin, Ras/mitogen-activated protein kinase (MAPK) and calcium signaling (Supplementary Table S6).

#### 2.1.4. Cluster-Specific Functional Characterization of LAP1-Interacting Proteins Through In Silico Analysis

In an effort to improve the functional characterization of LAP1-interacting proteins, we decided to investigate the interconnections between them, as this could allow uncovering multiprotein complexes within the LAP1 interactome and to ascribe more specific biological roles to the interaction partners and, in turn, to LAP1, thus complementing the initial generalized analysis. Upon utilizing the STRING database [34] to obtain the complete PPI network encompassing the 375 LAP1 interactors, where 271 proteins were linked by physical associations (Supplementary Figure S1), the Markov clustering (MCL) algorithm was used to rearrange this highly complex network into subgroups of physically connected proteins, leading to the retrieval of 44 clusters (Supplementary Figure S2; Supplementary Table S7). Then, a targeted in silico analysis of each cluster was carried out by inspecting the over-represented terms of GO categories and signaling pathways provided by the STRING database [34]; in two cases, different clusters were analyzed together—(i) clusters 4 and 12 as well as (ii) clusters 11 and 23—, given the physical interaction (Supplementary Figure S2) and functional association (i.e., inclusion in the same protein class; Supplementary Table S5) existing among some proteins of those clusters. Noteworthy, these functional enrichment analyses centered on protein clusters (Supplementary Tables S8–S47) offered valuable information regarding the functional features of LAP1-binding partners; in fact, additional physiological roles were highlighted, such as DNA repair (cluster 1) (Supplementary Table S8), ER-associated degradation (ERAD; clusters 3, 4 and 12) (Supplementary Tables S10 and S11), *N*-linked protein glycosylation (cluster 7) (Supplementary Table S14), RNA splicing and decay (clusters 11 and 23) (Supplementary Table S18), cilium assembly (cluster 15) (Supplementary Table S21), mitochondrial organization and function (clusters 17, 19, 31 and 36) (Supplementary Tables S23, S25, S34 and S39), immune response (cluster 26) (Supplementary Table S31) and reactive oxygen species (ROS) detoxification (cluster 32) (Supplementary Table S35).

**Figure 4:**
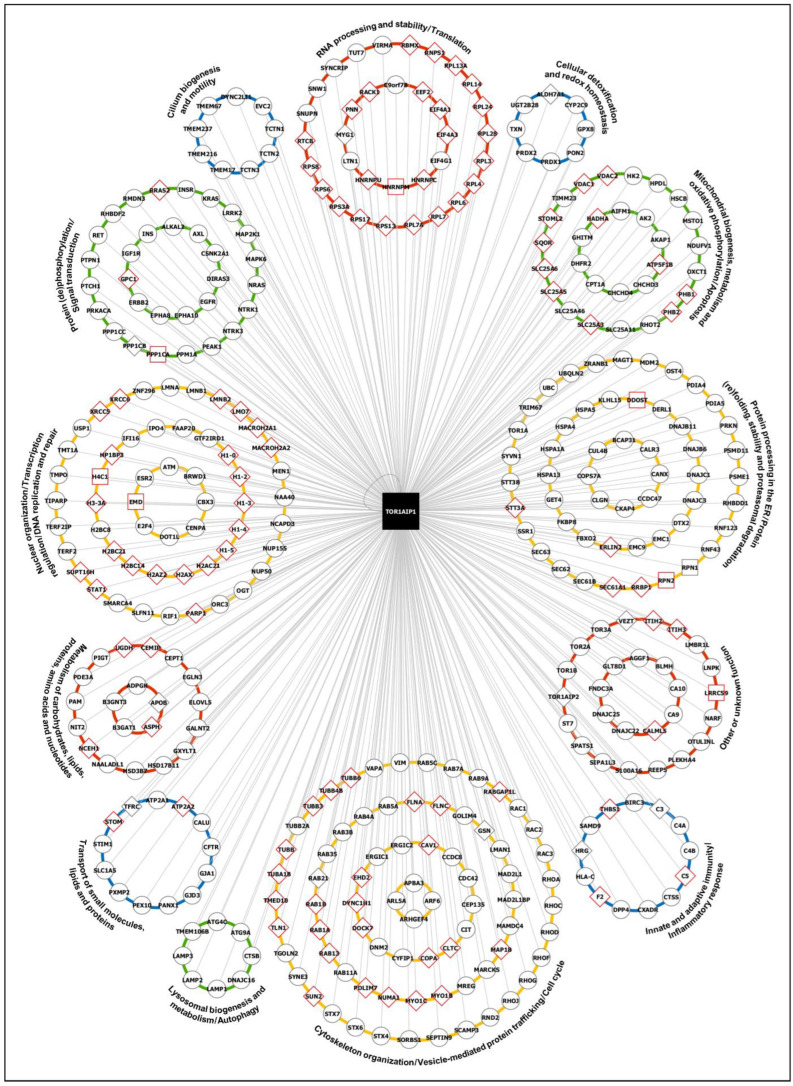
LAP1 (*TOR1AIP1*) protein interactome. The network, which comprises LAP1-interacting proteins (identified by gene name) retrieved from online databases and/or detected by LC–MS/MS in control fibroblasts, was constructed using the Cytoscape software [36]. The list of 375 LAP1-interacting proteins was organized into 13 functional groups (as indicated in the figure). Nodes with a circle shape (○) or diamond shape (◊) represent LAP1-interacting proteins found exclusively in online databases or control fibroblasts, respectively, while nodes with a square shape (□) symbolize LAP1-interacting proteins identified in both online databases and control fibroblasts. Nodes with a red contour line indicate LAP1-interacting proteins detected by LC–MS/MS in control fibroblasts but not in LAP1 E482A fibroblasts. Edges denote interactions between LAP1 and its binding partners. DNA, deoxyribonucleic acid; ER, endoplasmic reticulum; LAP1, lamina-associated polypeptide 1; LC–MS/MS, liquid chromatography coupled to tandem mass spectrometry; RNA, ribonucleic acid.

Altogether, the findings derived from the global and cluster-specific characterization of the LAP1 interactome reinforce the idea that LAP1 might be a functionally versatile protein. To better demonstrate this, we evaluated the overall GO annotations with the g:Profiler web tool [35] and/or published literature related with the LAP1-interacting proteins and subsequently organized them into functional groups; namely, (i) Nuclear organization/Transcription regulation/DNA replication and repair; (ii) RNA processing and stability/Translation; (iii) Protein processing in the ER/Protein (re)folding, stability and proteasomal degradation; (iv) Cellular detoxification and redox homeostasis; (v) Mitochondrial biogenesis, metabolism and oxidative phosphorylation/Apoptosis; (vi) Cytoskeleton organization/Vesicle-mediated protein trafficking/Cell cycle; (vii) Lysosomal biogenesis and metabolism/Autophagy; (viii) Innate and adaptive immunity/Inflammatory response; (ix) Cilium biogenesis and motility; (x) Protein (de)phosphorylation/Signal transduction; (xi) Metabolism of carbohydrates, lipids, proteins, amino acids and nucleotides; (xii) Transport of small molecules, lipids and proteins; and (xiii) Other or unknown function (Figure 4; Supplementary Table S48); for simplification, these functional protein groups will be referred hereafter as (i) DNA-, (ii) RNA-, (iii) proteostasis-, (iv) oxidative stress-, (v) mitochondria-, (vi) cytoskeleton-, (vii) lysosome-, (viii) immunity-, (ix) cilium-, (x) signaling-, (xi) metabolism- and (xii) transport-related groups. As Figure 4 clearly shows, LAP1 might be a central actor in regulating the global functioning of human cells by associating with proteins that exhibit such diverse physiological roles. Furthermore, it should be pointed out that the above results revealing that some LAP1 interactors participate in DDR/DNA repair (Supplementary Table S8), translation (Supplementary Table S9), protein folding/degradation (Supplementary Tables S10, S11 and S44) and antioxidant response (Supplementary Table S35) are quite interesting, since these biological processes and various proteins functionally associated to them were found deregulated in LAP1-deficient patient’s cells carrying the LAP1 E482A mutation [32], which supports the proposed involvement of LAP1 in these cellular activities. Accordingly, this motivated us to conduct a final integrative analysis, presented in the next subsection, that combined two major proteomic datasets: (i) LAP1-binding partners (Supplementary Tables S1–S3); and (ii) differentially expressed proteins previously identified in LAP1 E482A fibroblasts (Supplementary Tables S1 and S2 in the study by Pereira et al. (2024) [32]).

### 2.2. Comprehensive In Silico Analysis of LAP1-Interacting Proteins and LAP1-Deficient Human Fibroblasts’ Differentially Expressed Proteins

#### 2.2.1. PPI Analysis

**Figure 5:**
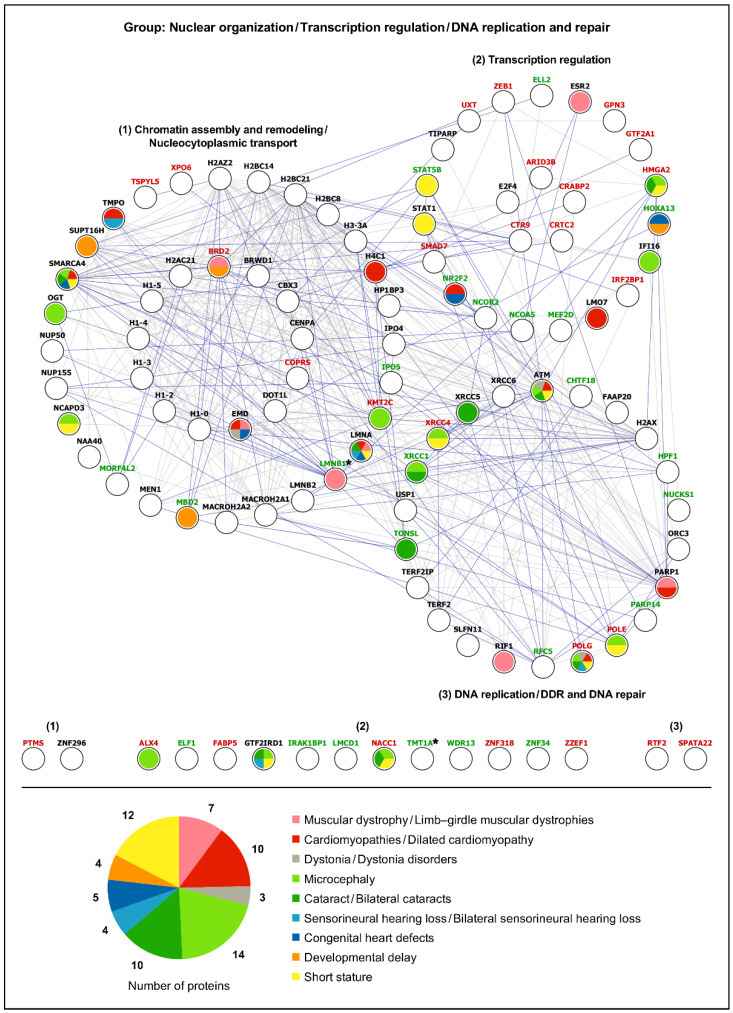
PPI and gene–disease association network of ‘Nuclear organization/Transcription regulation/DNA replication and repair’ functional protein group. The network comprises differentially expressed proteins found in LAP1 E482A fibroblasts [32] as well as LAP1-interacting proteins (identified by gene name). The PPI data were retrieved from the STRING database [34] and the PPI network was constructed using the Cytoscape software [36]. Nodes with a black label represent LAP1-interacting proteins, while nodes with a green or red label symbolize, respectively, upregulated or downregulated proteins (the black asterisk following the name indicates deregulated proteins that are LAP1 interactors). Blue edges denote physical/functional associations between LAP1-interacting proteins and LAP1 E482A fibroblasts’ differentially expressed proteins, whereas grey edges correspond to associations between proteins of the same proteomic dataset. The gene–disease association data were retrieved from the DisGeNET online platform [37]. The different colors filling the nodes’ interior represent specific diseases/pathological phenotypes (as indicated in the figure) associated to the proteins. The total number of proteins with known associations to each LAP1-related disease/pathological phenotype is also shown. DDR, DNA damage response; DNA, deoxyribonucleic acid; LAP1, lamina-associated polypeptide 1; PPI, protein–protein interaction.

**Figure 6:**
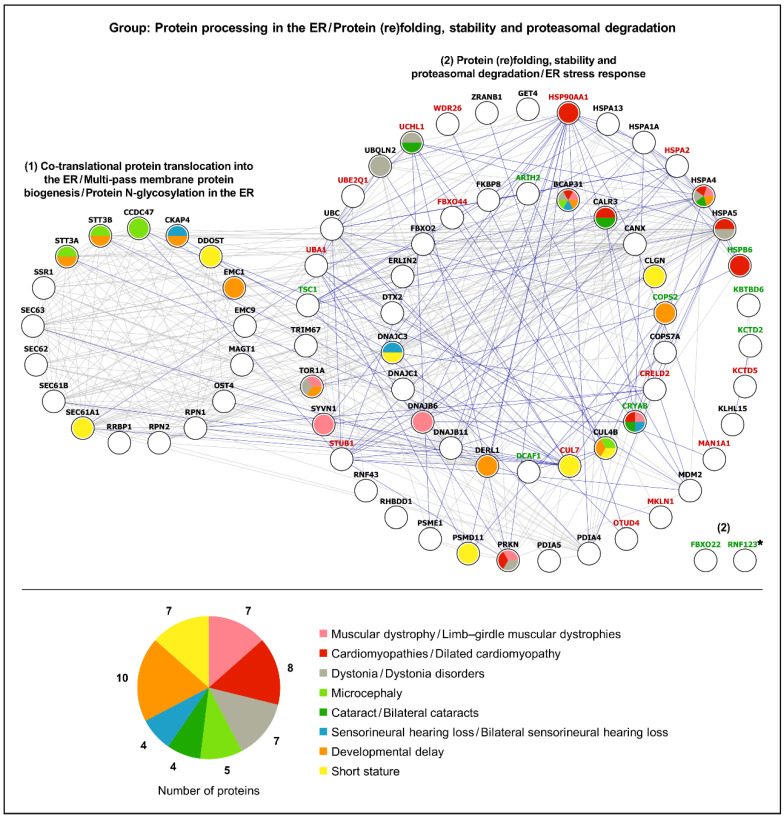
PPI and gene–disease association network of ‘Protein processing in the ER/Protein (re)folding, stability and proteasomal degradation’ functional protein group. The network comprises differentially expressed proteins found in LAP1 E482A fibroblasts [32] as well as LAP1-interacting proteins (identified by gene name). The PPI data were retrieved from the STRING database [34] and the PPI network was constructed using the Cytoscape software [36]. Nodes with a black label represent LAP1-interacting proteins, while nodes with a green or red label symbolize, respectively, upregulated or downregulated proteins (the black asterisk following the name indicates deregulated proteins that are LAP1 interactors). Blue edges denote physical/functional associations between LAP1-interacting proteins and LAP1 E482A fibroblasts’ differentially expressed proteins, whereas grey edges correspond to associations between proteins of the same proteomic dataset. The gene–disease association data were retrieved from the DisGeNET online platform [37]. The different colors filling the nodes’ interior represent specific diseases/pathological phenotypes (as indicated in the figure) associated to the proteins. The total number of proteins with known associations to each LAP1-related disease/pathological phenotype is also shown. ER, endoplasmic reticulum; LAP1, lamina-associated polypeptide 1; PPI, protein–protein interaction.

Using the analytical approach mentioned in Section 2.1.4, the list of 386 differentially expressed proteins detected in LAP1 E482A versus control fibroblasts [32] was organized into the same 13 functional protein groups (Supplementary Table S49) as performed initially for the list of LAP1 interactors (Supplementary Table S48). Afterwards, we performed a bioinformatic analysis of each group to explore both physical and functional associations of the deregulated proteins uncovered in LAP1 E482A fibroblasts with the LAP1-interacting proteins (Figure 5 and Figure 6; Supplementary Figures S3–S13). Based on the PPI data retrieved from the STRING database [34], it was possible to perceive that there exists ample interconnectivity between the LAP1 E482A fibroblasts’ differentially expressed proteins and the interaction partners of LAP1; this appears to occur particularly in the DNA- (Figure 5), RNA- (Supplementary Figure S3), proteostasis- (Figure 6), cytoskeleton- (Supplementary Figure S6) and signaling-related (Supplementary Figure S10) functional groups. Curiously, 12 LAP1 interactors were among the proteins exhibiting an altered expression pattern in LAP1 E482A fibroblasts (Figure 5 and Figure 6; Supplementary Figures S6–S8, S12 and S13), indicating that their deregulation could be directly correlated with LAP1 depletion; these include, for example: really interesting new gene (RING) finger protein 123 (RNF123), an E3 ubiquitin–protein ligase; the small G proteins Rab3B and Rab5C; filamins A and C, two actin-binding proteins; and the NE protein lamin B1. In an analogous way, it seems conceivable the idea that the altered abundance levels of other proteins, especially those physically/functionally connected to LAP1-binding partners (Figure 5 and Figure 6; Supplementary Figures S3–S8 and S10–S13), described in these patient’s cells could also be an indirect consequence of LAP1 deficiency. As such, envisioning a scenario wherein LAP1 forms physiologically relevant complexes with its interactors, which, in turn, associate with some of the proteins deregulated in LAP1 E482A fibroblasts, we hypothesized that the dysfunction of these different proteins could culminate in overlapping pathological phenotypes; this prompted us to carry out a gene–disease association analysis.

#### 2.2.2. Gene–Disease Association Analysis

In order to decipher if the differentially expressed proteins detected in LAP1 E482A fibroblasts and the LAP1-interacting partners might be implicated in pathologies similar to the rare LAP1-associated nuclear envelopathies, the DisGeNET online platform [37] was used. Considering that *TOR1AIP1* mutations leading to LAP1 deficiency have been shown to cause limb–girdle muscular dystrophy, dilated cardiomyopathy, dystonia, congenital myasthenic syndrome and a complex multisystemic disorder (characterized by microcephaly, developmental delay, bilateral cataracts, bilateral sensorineural hearing loss, congenital heart defects and growth retardation/short stature, among other variable clinical manifestations) [16,23,24,25,26,27,28,29,30,31], we restricted this in silico analysis to gene–disease associations between the proteins of each functional group and the referred diseases/pathological phenotypes (Supplementary Tables S50–S62), as explicated in the Materials and Methods section (see Section 4.6.2). The results revealed that a large number of the analyzed proteins are linked to cardiomyopathies/dilated cardiomyopathy (85 proteins), short stature (76 proteins), microcephaly (63 proteins) and cataract/bilateral cataracts (58 proteins) (Supplementary Figure S14). Additionally, a lower but still considerable portion of these proteins are associated with developmental delay (40 proteins), sensorineural hearing loss/bilateral sensorineural hearing loss (36 proteins), muscular dystrophy/limb–girdle muscular dystrophies (35 proteins), dystonia/dystonia disorders (32 proteins) and congenital heart defects (24 proteins), whereas only a few proteins are involved in congenital myasthenic syndromes (5 proteins) (Supplementary Figure S14). When establishing a comparison between the different functional protein groups, we noted that, in general, these exhibit pathological links to the various clinical conditions under analysis (Figure 5 and Figure 6; Supplementary Figures S3–S13; Supplementary Tables S50–S62), with a substantial part of the gene–disease associations being ascribed to the cytoskeleton- (Supplementary Figure S6; Supplementary Table S55), DNA- (Figure 5; Supplementary Table S50), signaling- (Supplementary Figure S10; Supplementary Table S59) and proteostasis-related (Figure 6; Supplementary Table S52) groups (87, 69, 61 and 52 associations, respectively). Contrasting with the more heterogeneous gene–disease associations observed in these functional groups, there is a predominance of cardiomyopathies among the pathologies linked to the immunity- (Supplementary Figure S8; Supplementary Table S57) and mitochondria-related (Supplementary Figure S5; Supplementary Table S54) groups.

Having verified that LAP1 interactors and LAP1 E482A fibroblasts’ deregulated proteins belonging to various functional groups are correlated with disorders that resemble those triggered by LAP1 dysfunction, we then focused on the DNA- and proteostasis-related groups. Special attention was given to these because it was recently found that LAP1 E482A fibroblasts present an abnormal increase in steady-state DNA damage levels and insoluble protein content comparatively to control fibroblasts and that the rescue of LAP1 protein expression helped in attenuating both the DNA damage and downregulation of the molecular chaperone heat shock protein (HSP) 90α family class A member 1 (HSP90α) in patient’s cells [32], suggesting that LAP1 could play a direct role in the maintenance of genome and proteome stability. The bioinformatic analysis of the DNA-related group, whose proteins regulate chromatin assembly and remodeling, nucleocytoplasmic transport and gene transcription as well as DNA replication and repair, retrieved numerous associations of its proteins mostly with microcephaly, short stature, cardiomyopathies, cataracts and muscular dystrophies (Figure 5; Supplementary Table S50). In the case of the proteostasis-related group, the results unveiled developmental delay, cardiomyopathies, dystonia, muscular dystrophies and short stature as the main pathologies linked to this group, which comprises proteins that control the translocation of nascent polypeptides into the ER, biogenesis of multi-spanning transmembrane proteins, *N*-glycosylation, protein (re)folding and proteasome-mediated proteolysis (Figure 6; Supplementary Table S52). Importantly, several proteins within these functional groups clearly stand out, due to being associated with, at least, half of the 10 diseases/pathological phenotypes of interest, namely: (i) ataxia–telangiectasia mutated (ATM), a DDR-linked protein kinase; (ii) the NE protein lamin A/C; (iii) switch defective/sucrose non-fermentable (SWI/SNF)-related, matrix-associated, actin-dependent regulator of chromatin subfamily A member 4 (SMARCA4), a chromatin remodeler; (iv) the mitochondrial DNA polymerase γ (Pol γ) catalytic subunit (Figure 5; Supplementary Table S50); (v) the ER chaperone B cell receptor-associated protein 31 (BCAP31); and (vi) heat shock protein family A member 4 (HSPA4), a cytosolic chaperone (Figure 6; Supplementary Table S52). With the exception of the downregulated protein Pol γ identified in LAP1 E482A fibroblasts, the remaining five proteins constitute LAP1 interactors, revealing that perturbations in the interaction of LAP1 with these partners might contribute considerably to the pathogenesis of *TOR1AIP1*-related nuclear envelopathies; hence, the physiological relevance of such LAP1-containing protein complexes merits further investigation.

## 3. Discussion

Cells rely on a complex interplay between proteins to accomplish their biological functions and maintain a living organism’s health. Mapping PPI networks is crucial to gain a deeper mechanistic understanding of how these cellular components work, which, in turn, can help to decipher the molecular basis of human diseases [38]. The INM protein LAP1 is critically important for normal cell functioning and survival, as proved by the exceptionally rare and pathogenic character of *TOR1AIP1* mutations causing the loss of LAP1B and/or LAP1C expression, along with the fact that these genetic variants are not found in the healthy population [16,23,24,25,26,28,29,30,31]. Such essentiality of LAP1 for the maintenance of cell homeostasis suggests that it might act in multiple PPI networks that regulate vital cellular activities. Therefore, profiling the protein interactome of LAP1 could be very useful to predict its physiological roles and respective mechanisms of action, which remain enigmatic.

In this study, we initially focused on the identification and functional characterization of LAP1-interacting proteins using different complementary strategies. First, 265 LAP1 interactors were identified by searching for binary PPIs of human/mouse LAP1 with human proteins in publicly available online databases (Supplementary Tables S1 and S2). In 2016, the in silico approach employed by Serrano et al. led to the detection of 41 LAP1-binding partners, including 36 human, two rodent and three viral interactors [22]. Comparing the output of these bioinformatic analyses, it becomes clear that the number of known LAP1-interacting proteins has grown substantially over the past years, many of which have been uncovered in large-scale human interactome projects. In view of that, we explored another screening methodology with potential to unveil additional interaction partners of LAP1 in human cells by performing an experimental analysis centered on the protein of interest. Using a LAP1-specific antibody to immunoprecipitate LAP1-containing protein complexes, it was possible to identify, by applying the LC–MS/MS technology, 110 novel LAP1 interactors in a control skin fibroblast cell line (Supplementary Table S3), allowing us to expand the LAP1 interactome dataset to 375 human proteins.

The ability of LAP1 to interact with a plethora of proteins reflects its functional versatility and further highlights the need to increase our knowledge about these PPIs to decode LAP1’s functioning. The interactomics data presented here can either set the basis for future investigations by aiding in the elaboration of research hypotheses or serve to consolidate prior findings. For example, the LAP1:torsinA complex is known to modulate the assembly and secretion of apolipoprotein B (apoB)-containing VLDLs from hepatocytes and it has been postulated that LAP1-dependent torsinA activity at the NE may be necessary for apoB lipidation and its folding and/or trafficking of this lipid-carrying protein [21]. Remarkably, apoB was detected as a new LAP1-interacting protein in our experiments (Supplementary Table S3), which strengthens the proposed model linking the LAP1:torsinA complex to apoB-mediated VLDL metabolism [21].

Since the biological significance of LAP1 binding to most of its partners is currently unclear, the in silico functional characterization of the latter emerges as a valuable tool to give initial clues about the most probable functions of LAP1. In agreement with the report by Serrano et al. [22], both global (Supplementary Table S6) and cluster-specific (Supplementary Tables S8–S47) enrichment analyses of GO, KEGG and Reactome terms assigned to LAP1 interactors reinforced the idea that LAP1 may operate in DDR, NE organization, mitotic cell cycle, protein folding, telomere maintenance and apoptosis. More significantly, our results brought to light a possible link between LAP1 and other previously unidentified biological processes, such as RNA splicing and decay, translation, protein processing in the ER, ERAD, ROS detoxification, mitochondrial biogenesis, vesicular transport, actin/microtubule cytoskeleton organization, autophagy, immune response and ciliogenesis. It is interesting to note that, by lying at the nucleocytoplasmic interface, LAP1 is at a unique position to regulate activities not only in the nucleus but in the cytoplasm as well, consistent with the bioinformatics data. Given the multifunctionality of the LAP1 interactome network, we sorted the interactors into 13 functional groups and noticed that the DNA-, proteostasis- and cytoskeleton-related groups together comprised almost half of LAP1-binding proteins (Figure 4; Supplementary Table S48). This seems to imply that LAP1 might have a more prominent role in the molecular mechanisms associated to these functional groups, like chromatin assembly and remodeling, DNA repair, co-translational protein translocation into the ER, *N*-glycosylation, protein (re)folding and proteasomal degradation, cytoskeleton dynamics and vesicle-mediated trafficking.

Overall, the growing repertoire of LAP1 interactors allied to their great functional diversity are strong indicators of the physiological importance of LAP1 in distinct cellular pathways. Accordingly, it is not surprising that LAP1 depletion can lead to pleiotropic pathological consequences, especially if it exerts a regulatory action on its binding partners. A major conclusion taken from our work is that the biological processes where many LAP1-interacting proteins intervene (Figure 4) are correlated with the molecular mechanisms deregulated in LAP1 E482A fibroblasts [32] and which, presumably, might also be dysfunctional in other patient-derived LAP1-deficient cell lines. An abnormal increase in DNA damage levels, protein synthesis rate, insoluble protein content and nuclear factor erythroid 2-related factor 2 (Nrf2)-induced antioxidant defense has been observed in LAP1 E482A versus control fibroblasts [32]. Having in mind that the LAP1 interactome network contains multiple DDR/DNA repair factors, 40S/60S ribosomal and other translational proteins, chaperones and components of the ubiquitin–proteasome system (UPS), oxidoreductases implicated in metabolic detoxification, as well as proteins required for mitochondrial integrity and respiratory chain activity (Figure 4), one can envision that such alterations in LAP1-depleted patient’s cells likely stem from the disrupted assembly of functional complexes between LAP1 and the referred proteins. As expected, our experiments revealed that most physiological LAP1 interactions identified in control fibroblasts (Figure 4; Supplementary Table S3) are not detected, under similar conditions, in LAP1 E482A fibroblasts (Figure 4; Supplementary Table S4).

To better examine how the disruption of LAP1-containing protein complexes can lead to human disease, we ultimately performed a combined analysis of the LAP1 interactors with the differentially expressed proteins found in LAP1 E482A fibroblasts [32]. The results evidenced numerous physical/functional relationships between the two proteomic datasets, including within the DNA- (Figure 5), proteostasis- (Figure 6) and cytoskeleton-related (Supplementary Figure S6) groups, suggesting that the interconnected LAP1-binding partners and LAP1 E482A fibroblasts’ deregulated proteins could jointly contribute to a shared function. Furthermore, the fact that the pathological phenotypes linked to several proteins from both datasets coincide with LAP1-related disorders (Figure 5 and Figure 6; Supplementary Figures S3–S13; Supplementary Tables S50–S62) hints at a physiologically important cooperation between LAP1, its interactors and those proteins that display an altered expression pattern in a context of LAP1 deficiency. As such, a more detailed investigation of how all of these proteins act in concert to control specific cellular activities is warranted to fully decipher the functional attributes of LAP1 and the disturbed mechanisms in *TOR1AIP1*-associated nuclear envelopathies. This knowledge could be fundamental to developing effective therapies for these devastating rare pathologies and, perhaps, could be helpful to target more common diseases as well.

Our study represents a significant advancement in understanding the LAP1 protein interactome and its biological properties. However, it also presents some limitations that should be addressed in future investigations. For instance, our experimental analysis of LAP1-interacting proteins was performed exclusively in human skin fibroblasts, but it would be relevant to further extend this analysis to other cell/tissue types to determine cell-/tissue-specific binding partners of LAP1, which could help dissecting the more specialized functions of this INM protein. Another limitation of this work includes the possibility of not detecting weak or transient protein interactions and, in light of the multiple biological processes to which LAP1 has been associated, it would be of interest to analyze its network of interactors in specific cellular contexts (e.g., upon induction of DNA damage), as some interactions might be triggered and/or stabilized by a particular stimulus. Moreover, to attest the reliability of our LC–MS/MS findings, it will be important to validate the putative novel LAP1-interacting proteins identified in control fibroblasts using alternative methodologies. It should be highlighted that we have already confirmed the LAP1:Ku70 and LAP1:H2AX interactions through immunoblotting analysis of LAP1 co-immunoprecipitates (Figure 2c). Additional candidate LAP1-interacting proteins identified in this study should be validated in future work and, ideally, the biological significance of the association between LAP1 and such binding partners should also be investigated.

In the following subsections, we provide some preliminary mechanistic insights into the putative biological roles of LAP1 in the maintenance of genome and proteome homeostasis through an integrative overview of the data presented in this study.

### 3.1. Spatial Genome Organization and Chromatin Remodeling

In the eukaryotic cell, the nuclear genome is non-randomly partitioned into various territories that differentially shape its transcriptional status. Typically, the less condensed, transcriptionally active euchromatin is located in the nuclear interior and underneath nuclear pore complexes (NPCs), while the more densely packed, transcriptionally silent heterochromatin is positioned around nucleoli and lining the NE between nuclear pores [39]. LAP1 is perfectly situated to control the organization of peripheral heterochromatin, like lamina-associated domains (LADs), which consist of genomic elements in an overall repressed state that are in contact with the nuclear lamina and INM [39]. Several lines of evidence appear to support this attractive hypothesis, as follows.

First, LAP1 serves as an INM adaptor for the nuclear lamina, as it has been shown to directly interact with both A- and B-type lamins [2,7] and be required for lamin A/C positioning in close proximity to the INM [26]. Considering this and the fact that lamin A/C is essential for heterochromatin tethering to the NE in differentiated cells [40], it is reasonable to propose that LAP1 may be an INM-resident functional partner of lamin A/C in organizing LADs.

Second, besides its physical linkage to the nuclear lamina, LAP1 may exhibit additional heterochromatin-anchoring properties by directly binding chromatin [2] and via its interactors menin, heterochromatin protein 1γ (HP1γ)—retrieved from online databases (Supplementary Table S1)— linker histones H1, as well as core histone variants macroH2A1 and macroH2A2—discovered in our experiments (Supplementary Table S3). These LAP1-interacting proteins can contribute to the repression of genomic loci by promoting the formation of heterochromatin-specific histone marks, namely di/trimethylation of histone H3 at Lys9 (H3K9me2/3) (e.g., menin [41], HP1γ [42,43] and histones H1 [44]), and to the immobilization of H3K9me3-modified heterochromatic domains at the nuclear periphery (e.g., histones macroH2A1 and macroH2A2 [45,46]).

Third, LAP1 may also have a role in heterochromatinization by interacting with proteins that modulate local chromatin compaction. Our experimental approach uncovered LAP1-binding partners that are known to induce the higher-order packaging of nucleosomes, such as linker histones H1 [44] and heterochromatin protein 1-binding protein 3 (HP1BP3) [47] (Supplementary Table S3).

Although future work will be needed to experimentally validate the hypothesized LAP1’s physiological functions, the presented data collectively suggest that LAP1, allied to its broad PPI network present at the INM–nuclear lamina–chromatin interface, may control chromatin dynamics via multiple mechanisms (e.g., epigenetic silencing of chromatin, heterochromatin sequestering within LADs and structural reorganization of nucleosomes) and, consequently, may affect gene expression. Quite impressively, the integrated bioinformatic analysis of interactomics and proteomics data demonstrated that, while LAP1 interactors are over-represented in the subgroup implicated in chromatin assembly and remodeling, the differentially expressed proteins identified in LAP1 E482A fibroblasts [32] clearly dominate in the subgroup involved in DNA transcription (Figure 5). One possible interpretation of these results is that the protein level changes in transcription factors and members of the RNA polymerase II transcription machinery (e.g., homeobox A13 (HOXA13), general transcription factor IIA subunit 1 (GTF2A1) and myocyte-specific enhancer factor 2D (MEF2D)) detected in these LAP1-deficient patient’s cells [32] could be secondary to the dysfunction of LAP1 and its numerous binding partners that jointly ensure a normal spatial genome architecture, as those discussed above. For instance, in the case of lamin A/C, mutations in the *LMNA* gene leading to a multisystemic premature aging disorder [48] or dilated cardiomyopathy [49] have been linked to altered deposition of repressive histone marks and loss of heterochromatin–nuclear lamina association, culminating in transcriptional misregulation. The fact that substantial overlap exists between lamin A/C- and LAP1-related nuclear envelopathies (Figure 5) could indicate that similar molecular mechanisms are deregulated in cells affected by *TOR1AIP1* mutations and is consistent with the perspective that LAP1 may act as a chromatin regulator, at least in part, by anchoring lamin A/C to the NE. Notably, increased chromatin condensation and heterochromatin detachment from the INM have been observed in skeletal muscle samples from *TOR1AIP1* patients with muscular dystrophy [23,29,30] and congenital myasthenic syndrome [16], respectively, thus strengthening the link between LAP1 and heterochromatinization.

### 3.2. DNA Repair

In the mammalian nucleus, the highly cytotoxic DNA double-strand breaks (DSBs) can be repaired by different pathways depending on their spatial positioning: DSBs arising in the nuclear interior or close to NPCs are repaired by non-homologous end joining (NHEJ) or homologous recombination (HR), while the repair of DSBs originated at LADs occurs via NHEJ or alternative end joining (alt-EJ) [50]. Previous studies from our group provided preliminary experimental evidence of LAP1 involvement in DSB repair [10,32]. In a first work addressing the interplay between LAP1 and TRF2, we showed that their interaction is stimulated after exposing cells to genotoxic agents and that LAP1 co-localizes with TRF2 and a DNA damage marker (i.e., histone variant H2AX phosphorylated at Ser139 (γ-H2AX)) in foci located at the NE and inside the nucleus [10]. Since TRF2 participates in NHEJ [51] and HR [52,53], we hypothesized that the presence of the LAP1:TRF2 complex in distinct nuclear regions could reflect the repair of intranuclear euchromatic DSBs and peripheral heterochromatic DSBs [10], though the second scenario seems more likely given LAP1’s main location in the perinuclear rim.

Further evidence of the physiological relevance of LAP1 in DSB repair comes from our finding of an inverse relationship between intracellular LAP1 protein levels and the content of unrepaired DNA lesions. In a recent report, we demonstrated that LAP1 E482A fibroblasts exhibit increased γ-H2AX levels at baseline as well as hypersensitivity to a genotoxic agent [32], suggestive of a delayed or insufficient capacity to properly repair DSBs in the absence of LAP1. In line with this, we were able to attenuate basal γ-H2AX levels in LAP1-deficient patient’s cells by restoring LAP1B and/or LAP1C protein expression [32], unveiling that both LAP1 isoforms can aid in the preservation of genome integrity in human cells.

Of particular significance, the LAP1 E482A fibroblasts’ proteome analysis uncovered a downregulation of X-ray repair cross-complementing protein 4 (XRCC4) [32], whose deficiency has been proven to lead to severely defective NHEJ-mediated DSB repair [54,55]. Therefore, it is conceivable that NHEJ impairment could contribute to DNA damage accumulation in LAP1 E482A fibroblasts [32] and that LAP1 might play a role in this DNA repair pathway. This novel hypothesis appears to be supported by the results of the present study, wherein Ku70 and Ku80 were identified as new LAP1 interactors (Supplementary Table S3). These proteins form the Ku heterodimer (Ku), a central DSB sensor in NHEJ that rapidly recognizes and binds broken DNA ends [56]. At the DSB site, Ku serves as a major hub that promotes the efficient recruitment of core NHEJ effectors, namely DNA ligase IV (Lig4) and its obligate binding partner XRCC4 [56,57], which potentiates the DNA end-joining activity of the XRCC4:Lig4 complex [57]. Given these physiological (i.e., LAP1 interaction with Ku70/Ku80 in control fibroblasts; Supplementary Table S3) and pathological (i.e., XRCC4 downregulation in LAP1 E482A fibroblasts [32]) associations with NHEJ, it is tempting to speculate that LAP1 might act as a positive regulator of the main DSB repair pathway in mammalian cells. Noteworthy, it has been documented that Ku80 is required for the active restriction of DSB mobility within the nucleus, preventing the inappropriate joining of broken DNA ends generated at different genomic loci that lead to mutagenic chromosomal translocations [58]. It can, thus, be hypothesized that the LAP1:Ku80 interaction could help immobilizing Ku-bound DSBs via physical linkage to the NE and allow for their repair at the original location, maintaining the proper association of DNA end pairs arising from the same DSB. In light of the recent observation of decreased *TOR1AIP1* expression levels in several cancer types [59], it will be of great interest to investigate the presumed function of LAP1 in heterochromatic DSB repair by NHEJ as well as the possible impact of LAP1 dysfunction on genomic (in)stability.

### 3.3. Protein Folding and Proteasomal Degradation

As pointed out earlier, the characterization of the LAP1 interactome evidenced that many LAP1-binding partners are involved in protein (re)folding and ubiquitin-dependent proteasomal degradation (Figure 6). This is compatible with the concept that LAP1 depletion may be accompanied by a loss of proteostasis, as suggested by the increase in protein aggregation seen in LAP1 E482A fibroblasts and further supported by the analysis of their proteome, wherein a deregulation of several foldase/holdase chaperones and UPS components was uncovered [32]. As such, it seems plausible that LAP1 might have an undefined physiological role contributing to the maintenance of protein homeostasis. While this predicted function of LAP1 still has to be experimentally determined, we postulate that it could be fulfilled via cooperation with torsinA.

The LAP1 E482A mutation and the best characterized torsinA ΔE302/303 mutation are predicted [2,60] and known [12,61], respectively, to negatively affect the LAP1:torsinA interaction and both have been associated to dystonia [24,62], emphasizing the biological relevance of LAP1-induced torsinA activation at the NE [12,13,19]. Importantly, the presence of INM blebs protruding into the NE lumen has been observed in torsinA [63,64] and LAP1 [64] loss-of-function animal models. Subsequent studies in torsin knockout cells revealed that such blebs sequester proteins conjugated to Lys48-linked ubiquitin—a signal for protein degradation by the 26S proteasome—, which might reflect a protein quality control (PQC) defect related with a failure to remove non-native proteins from the NE that, in turn, impedes their proteasomal destruction [65,66]. Taking into account that torsinA has already been reported to participate in ERAD, wherein it enhances the retro-translocation to the cytosol of ER substrates targeted for UPS-regulated proteolysis [67], it can be envisaged that torsinA and its cofactor LAP1 could interact at the NE to form an active ATPase machine that modulates INM protein turnover through an ERAD-like mechanism (i.e., INM-associated degradation (INMAD)). Notwithstanding this is a hypothetical scenario, it is interesting to note that the ATPase p97/valosin-containing protein (VCP), a known functional partner of torsinA in the canonical ERAD pathway [67], has also been implicated in the degradation of INM-localized lamin B receptor (LBR) mutants mediated by the nuclear UPS [68] as well as of the endogenous INM protein Sad1/UNC84 homology domain-containing protein 2 (SUN2) [69]. Hence, one appealing possibility is that the LAP1:torsinA complex may work together with VCP as part of a PQC system operating at the NE. Another potentially significant protein to consider in this context could be the E3 ubiquitin–protein ligase RNF123, a LAP1 interactor (Supplementary Table S2) that was found to be deregulated in LAP1 E482A fibroblasts [32]. RNF123 has been described to mediate the ubiquitination and consequent proteasomal degradation of NE and NE-associated proteins (e.g., lamin B1 and lamina-associated polypeptide 2α (LAP2α)) [70], indicating that it might function in NE quality control. Intriguingly, while it has previously been shown that RNF123 overexpression results in reduced lamin B1 protein levels [70], LAP1 E482A fibroblasts exhibit an upregulation of both RNF123 and lamin B1 [32], raising the question of whether RNF123 activity might be altered in LAP1-deficient cells.

In essence, there is growing evidence that perturbations in protein homeostasis at the NE may underlie the pathogenesis of LAP1-/torsinA-related dystonia disorders. Since the cellular components responsible for INMAD in mammalian cells have not been entirely deciphered [71], it would be important to explore if the LAP1:torsinA complex is involved in this mechanism that helps preserving a functional INM proteome.

## 4. Materials and Methods

### 4.1. Antibodies

The following primary antibodies were used for the detection of human target proteins by immunoblotting (IB) and/or co-immunoprecipitation (co-IP): rabbit polyclonal anti-LAP1 (Goodchild and Dauer [11]; 1:20,000 for IB; 0.2 µL per 2000 µg of protein for co-IP); mouse monoclonal anti-Ku70 (Santa Cruz Biotechnology (sc-5309), Heidelberg, Germany; 1:1000 for IB); mouse monoclonal anti-emerin (Developmental Studies Hybridoma Bank (MANEM1), Iowa City, IA, United States of America (USA); 1:90 for IB); and mouse monoclonal anti-histone variant H2AX (Santa Cruz Biotechnology (sc-517336); 1:500 for IB). In addition, horseradish peroxidase (HRP)-conjugated mouse anti-rabbit TrueBlot IgG (Rockland (18-8816-33), Limerick, PA, USA; 1:1000) and HRP-conjugated horse anti-mouse IgG (Cell Signaling Technology (7076), Leiden, The Netherlands; 1:10,000) were utilized as secondary antibodies for immunoblotting.

### 4.2. Human Cell Lines

Patient-derived LAP1 E482A skin fibroblast and age-/gender-matched control skin fibroblast cell lines, which had been established from human donors as previously described by Dorboz et al. [24], were made available for and used in the present study.

### 4.3. Cell Culture Procedures

Human fibroblast cell lines were cultured in Dulbecco’s modified Eagle medium (DMEM; Gibco, Thermo Fisher Scientific, Waltham, MA, USA) supplemented with 15% fetal bovine serum (FBS; Gibco) and 1% penicillin/streptomycin (Gibco). Cells were maintained at 37 °C in a humidified atmosphere with 5% CO_2_ and subcultured when a confluency of 80–90% was achieved.

LAP1 E482A and control fibroblasts from identical cell passages, which did not exceed cell passage 15, were used in all experiments. For co-immunoprecipitation, both cell lines were grown in standard conditions in 100 mm culture dishes until reaching 80–90% of confluency, after which cells were used in LAP1 co-immunoprecipitation assays (see Section 4.4).

### 4.4. Co-Immunoprecipitation

Human fibroblasts were scraped into 1× phosphate buffered saline (PBS) and cell pellets obtained by centrifugation were then resuspended in ice-cold 3-((3-cholamidopropyl) dimethylammonio)-1-propanesulfonate (CHAPS) lysis buffer (50 mM Tris-HCl, pH = 8; 120 mM sodium chloride (NaCl); 4% CHAPS) supplemented with phosphatase and protease inhibitors (1 mM phenylmethylsulfonyl fluoride (PMSF); 1× Halt protease and phosphatase inhibitor cocktail (Thermo Scientific, Thermo Fisher Scientific, Waltham, MA, USA)). Upon incubation at 4 °C during 30 min, cell lysates were sonicated on ice for 5 s (0.5 cycles, 60% amplitude) and total protein concentration was measured using the Pierce bicinchoninic acid (BCA) protein assay kit (Thermo Scientific), according to the manufacturer’s instructions. Dynabeads Protein G (Invitrogen, Thermo Fisher Scientific, Waltham, MA, USA) were firstly washed with ice-cold 3% bovine serum albumin (BSA)/1× PBS and subsequently cross-linked to a LAP1-specific primary antibody (see Section 4.1) or control rabbit IgG (Invitrogen) for 2 h at 4 °C. For each fibroblast cell line, cell lysate samples containing 2000 µg of protein were subjected to pre-clearance with Dynabeads Protein G for 1 h at 4 °C, followed by incubation with Dynabeads Protein G–antibody (or control IgG) conjugates at 4 °C overnight. The resulting LAP1 co-immunoprecipitates were either processed for LC–MS/MS analysis (see Section 4.5) or resolved by sodium dodecyl sulfate–polyacrylamide gel electrophoresis (SDS–PAGE) and analyzed by immunoblotting (see Section 4.7).

### 4.5. LC–MS/MS

For the LC–MS/MS analysis of LAP1-interacting proteins in control and LAP1 E482A fibroblasts, Dynabeads Protein G used in the co-immunoprecipitation assays (see Section 4.4) were cleaned with 200 mM ammonium bicarbonate and resuspended in 6 M urea/200 mM ammonium bicarbonate. Samples were then reduced with dithiothreitol (DTT; 30 nmol) at 37 °C for 1 h, alkylated in the dark with iodoacetamide (60 nmol) at 25 °C for 30 min and diluted to 1 M urea with 200 mM ammonium bicarbonate for digestion with trypsin (Promega, Madison, WI, USA; 1 µg) at 37 °C for 8 h. Afterwards, peptide mixes were acidified with formic acid and desalted with a MicroSpin C18 column (The Nest Group, Ipswich, MA, USA), being subsequently analyzed by LC–MS/MS.

Three replicates of each experimental condition (control IgG and LAP1 co-immunoprecipitation) obtained from each fibroblast cell line were analyzed using an Orbitrap Fusion Lumos mass spectrometer (Thermo Fisher Scientific) coupled to an EASY-nLC 1200 nanoflow liquid chromatography system (Thermo Fisher Scientific). Peptides were loaded directly onto the analytical column and separated by reversed-phase chromatography using a 50 cm column with an inner diameter of 75 µm, packed with 2 µm C18 particles (Thermo Fisher Scientific). Chromatographic gradients started at 95% buffer A (0.1% formic acid in water)/5% buffer B (0.1% formic acid in 80% acetonitrile) with a flow rate of 300 nL/min and gradually increased to 25% buffer B/75% buffer A in 52 min and then to 40% buffer B/60% buffer A in 8 min. After each analysis, the column was washed with 100% buffer B for 10 min.

The mass spectrometer was operated in the positive ionization mode, with the nano-spray voltage set at 2.4 kV and the source temperature at 305 °C. The acquisition was performed in the data-dependent acquisition (DDA) mode and full mass spectrometry (MS) scans, with 1 microscan at a resolution of 120,000, were used over an *m/z* range of 350–1400, with detection in the Orbitrap mass analyzer. The auto gain control (AGC) was set to ‘standard’ and the injection time to ‘auto’. In each cycle of DDA analysis, following each survey scan, the most intense ions above a threshold ion count of 10,000 were selected for fragmentation. The number of selected precursor ions for fragmentation was determined by the ‘Top Speed’ acquisition algorithm and a dynamic exclusion of 60 s. Fragment ion spectra were produced via high-energy collision dissociation (HCD) at normalized collision energy of 28% and acquired in the Ion Trap mass analyzer. The AGC and injection time were set to ‘standard’ and ‘dynamic’, respectively, and an isolation window of 1.4 *m/z* was used. Digested BSA MS standard (New England Biolabs, Ipswich, MA, USA) was analyzed between each sample to avoid sample carryover and to assure stability of the instrument. The QCloud quality control system [72,73] was used to control the instrument’s longitudinal performance during the LC–MS/MS experiments.

The acquired spectra were analyzed using the Proteome Discoverer software suite (version 2.5; Thermo Fisher Scientific) and the Mascot search engine (version 2.6; Matrix Science, London, United Kingdom (UK) [74]). LC–MS/MS data were searched against a Swiss-Prot human database (as in April 2022, 20,401 entries) plus a list of common contaminants [75] and all the corresponding decoy entries. For peptide identification, a precursor ion mass tolerance of 7 ppm was used for MS1 level, trypsin was chosen as enzyme and up to three missed cleavages were allowed. The fragment ion mass tolerance was set to 0.5 Da for MS2 spectra. The oxidation of methionine and N-terminal protein acetylation were used as variable modifications, while carbamidomethylation on cysteines was set as a fixed modification. The false discovery rate (FDR) in peptide identification was set to a maximum of 1%. The SAINTexpress algorithm [76] was used to score PPIs as well as to calculate the protein fold change (ratio of protein abundance between LAP1 co-immunoprecipitation samples and control IgG co-immunoprecipitation samples) and corresponding *p*-value. The raw MS proteomics data have been deposited to the PRoteomics IDEntifications (PRIDE) repository [77] with the dataset identifier PXD056445.

### 4.6. Bioinformatic Analysis

Two main bioinformatic analyses were performed: (i) a functional enrichment analysis of LAP1-interacting proteins identified by LC–MS/MS in this work and/or collected from online databases (see Section 4.6.1); and (ii) PPI and gene–disease association analyses integrating the referred LAP1 interactors and the differentially expressed proteins previously found in LAP1 E482A versus control fibroblasts [32] (see Section 4.6.2).

#### 4.6.1. Functional Enrichment Analysis of LAP1-Interacting Proteins

Following the LC–MS/MS analysis of the proteins that were co-immunoprecipitated with LAP1 in human fibroblasts (628 and 530 proteins with a positive fold change identified in control and LAP1 E482A fibroblasts, respectively), two criteria were used to select the relevant LAP1-interacting proteins in each fibroblast cell line: (i) protein fold change-associated *p*-value < 0.1 and (ii) SAINT score ≥ 0.7. Based on this, 118 and 15 proteins that co-immunoprecipitated with LAP1 in control and LAP1 E482A fibroblasts, respectively, were selected as LAP1 interactors for the bioinformatic analysis.

To identify previously known LAP1-binding partners, a search at several online public databases/web services was additionally conducted (all accessed on November 2023); these included: National Center for Biotechnology Information (NCBI) [78]; BioGRID (version 4.4.227) [79]; Human Integrated Protein–Protein Interaction rEference (HIPPIE; version 2.3) [80]; IntAct (version 1.0.4) [81]; mentha [82]; I2D (version 2.9) [83]; and Proteomics Standard Initiative Common QUery InterfaCe (PSICQUIC) View (version 1.6.1) [84]. The search focused on the interactors of human LAP1 protein (UniProt accession: Q5JTV8) and mouse Lap1 protein (UniProt accession: Q921T2), among which only human interacting proteins with experimentally detected PPIs were considered for subsequent analysis. Using this approach and also by consulting a prior in silico study that characterized the mammalian LAP1 interactome [22], 232 and 35 human protein interactors of human LAP1 and mouse Lap1, respectively, were selected for the bioinformatic analysis.

After combining the two datasets of LAP1-interacting proteins (i.e., those identified experimentally by LC–MS/MS in control fibroblasts and those retrieved from online databases) and excluding the duplicated ones, a final list of 375 LAP1 interactors was obtained and further analyzed using bioinformatics tools. Firstly, the PANTHER online resource (version 18.0; accessed on November 2023) [33] was used for the characterization of protein class categories. Next, the STRING database (version 12.0; accessed on November 2023) [34] was utilized to perform a functional enrichment analysis of GO biological process, GO molecular function, GO cellular component, KEGG pathways and Reactome pathways annotations, with the human whole genome being chosen as the reference list for the statistical analysis of enriched terms.

In addition to this global functional characterization extending to the full list of LAP1-interacting proteins, a cluster-specific bioinformatic analysis was posteriorly carried out using the STRING database (version 12.0; accessed on December 2023) [34]. A general PPI network comprising all LAP1 interactors was initially constructed by selecting the physical network subtype (experiments and databases as active interaction sources; minimum interaction score of 0.4). Then, by applying the MCL algorithm (inflation parameter of 1.8), the original PPI network was subdivided into clusters containing proteins linked by physical associations, after which each protein cluster was subjected to a functional enrichment analysis of GO categories and biological pathways, as described above. The Cytoscape software (version 3.10.1) [36] was posteriorly used for the graphical representation of the PPI networks retrieved from the STRING database [34] (i.e., general PPI network comprising all LAP1-interacting proteins and PPI subnetworks depicting protein clusters).

#### 4.6.2. PPI and Gene–Disease Association Analyses of LAP1-Interacting Proteins and LAP1 E482A Fibroblasts’ Differentially Expressed Proteins

Prior to analysis, the list of 375 LAP1 interactors generated in the present work (see Section 4.6.1) and the list of 386 differentially expressed proteins that had been identified by LC–MS/MS in LAP1-deficient fibroblasts carrying the LAP1 E482A mutation [32] were organized into different functional groups, designated as (i) Nuclear organization/Transcription regulation/DNA replication and repair; (ii) RNA processing and stability/Translation; (iii) Protein processing in the ER/Protein (re)folding, stability and proteasomal degradation; (iv) Cellular detoxification and redox homeostasis; (v) Mitochondrial biogenesis, metabolism and oxidative phosphorylation/Apoptosis; (vi) Cytoskeleton organization/Vesicle-mediated protein trafficking/Cell cycle; (vii) Lysosomal biogenesis and metabolism/Autophagy; (viii) Innate and adaptive immunity/Inflammatory response; (ix) Cilium biogenesis and motility; (x) Protein (de)phosphorylation/Signal transduction; (xi) Metabolism of carbohydrates, lipids, proteins, amino acids and nucleotides; (xii) Transport of small molecules, lipids and proteins; and (xiii) Other or unknown function. This functional categorization of the aforementioned proteins was performed following an analysis of the respective GO annotations using the g:Profiler web tool (version e111_eg58_p18_30541362; accessed on February–March 2024) [35], complemented by a general search of the published literature at PubMed (accessed on February–April 2024) [85].

Once the above functional protein groups were established, the STRING database (version 12.0; accessed on April 2024) [34] was employed to assess both functional and physical associations between the LAP1 E482A fibroblasts’ deregulated proteins and the LAP1-interacting proteins belonging to each group, which was accomplished by choosing the full network subtype (experiments, databases, text mining, co-expression, neighborhood, co-occurrence and gene fusion as active interaction sources; minimum interaction score of 0.4). In turn, a bioinformatic gene–disease association analysis of each functional group was carried out using the DisGeNET online platform (version 7.0; accessed on April 2024) [37]. This analysis focused solely on diseases/pathological phenotypes similar to those that have already been associated to *TOR1AIP1* mutations; as such, a filter was applied to the ‘Disease’ field to retrieve only those proteins with known associations to the following diseases/pathological phenotypes of interest: (i) Muscular dystrophy (CUI: C0026850); (ii) Muscular dystrophies, limb-girdle (CUI: C0686353); (iii) Cardiomyopathies (CUI: C0878544); (iv) Cardiomyopathy, dilated (CUI: C0007193); (v) Dystonia (CUI: C0013421); (vi) Dystonia disorders (CUI: C0393593); (vii) Myasthenic syndromes, congenital (CUI: C0751882); (viii) Microcephaly (CUI: C0025958); (ix) Cataract (CUI: C0086543); (x) Bilateral cataracts (CUI: C0521707); (xi) Sensorineural hearing loss (CUI: C0018784); (xii) Sensorineural hearing loss, bilateral (CUI: C0452138); (xiii) Congenital heart defects (CUI: C0018798); (xiv) Developmental delay (CUI: C0424605); and (xv) Short stature (CUI: C0349588). Lastly, based on the PPI data retrieved from the STRING database [34], as well as the gene–disease association data collected from the DisGeNET platform [37], the Cytoscape software (version 3.10.1) [36] was used to graphically illustrate the PPI networks representing the functional groups of proteins and respective associated disorders.

### 4.7. SDS–PAGE and Immunoblotting

For the immunoblotting analysis of LAP1 interactors in control and LAP1 E482A fibroblasts, protein immunoprecipitates obtained from LAP1 co-immunoprecipitation assays (see Section 4.4) were eluted from Dynabeads Protein G by boiling in 1× loading buffer (62.5 mM Tris, pH = 6.8; 2% SDS; 10% glycerol; 5% β-mercaptoethanol; 0.0025% bromophenol blue) at 90 °C for 10 min. Immunoprecipitated proteins were separated on a 10% polyacrylamide gel by SDS–PAGE, which was followed by electrophoretic transfer onto nitrocellulose membranes (0.2 µm pore size; GE Healthcare, Buckinghamshire, UK) and posterior immunological detection of target proteins. After initial blocking in 5% BSA/1× Tris-buffered saline with 0.1% Tween-20 (TBS-T) for 3 h, membranes were incubated with specific primary antibodies against LAP1 and its binding partners Ku70, emerin and H2AX (see Section 4.1) in 3% BSA/1× TBS-T for 2 h, with subsequent overnight incubation at 4 °C. Next, membranes were incubated with HRP-linked secondary antibodies (see Section 4.1) in 5% fat-free dry milk/1× TBS-T for 1 h and scanned in a ChemiDoc imaging system (Bio-Rad, Hercules, CA, USA) to detect protein bands by enhanced chemiluminescence (ECL).

### 4.8. Statistical Analysis

The functional enrichment analyses of LAP1-interacting proteins were carried out using the STRING database [34], wherein the FDR was corrected for multiple testing within each category by applying the Benjamini–Hochberg procedure. Enriched terms were considered as those with a *p*-value < 0.05.

## 5. Conclusions

LAP1 is emerging as an important signaling node at the NE. We recently showed that LAP1 depletion may affect global cell functioning by leading to the deregulation of several biological processes, such as DNA repair, translation, proteostasis and oxidative stress response [32], revealing that LAP1 might have broad functional properties. In the present study, adding to the numerous LAP1-binding proteins already described in databases, we identified many novel interaction partners using a LAP1-specific proteomics-based methodology. This work clearly illustrates the complexity of the PPI network of LAP1, which comprises proteins involved, for instance, in chromatin remodeling, DNA repair, RNA splicing, protein synthesis, folding and turnover, cytoskeleton dynamics and mitochondrial biogenesis, thus explaining the multifunctional nature of this NE protein. Through a comprehensive analysis, we uncovered relevant functional connections between LAP1 interactors and deregulated proteins detected in LAP1-deficient patient’s cells that allowed shedding light on potential roles of LAP1 in heterochromatin organization, NHEJ-mediated DSB repair and INMAD. While additional studies will be required to test these new ideas, our data provide compelling evidence of the physiological significance of LAP1 and a strong motivation to dissect its mechanisms of action in pivotal biological processes like DNA repair, as their investigation could clarify the pathological mechanisms contributing not only to the rare *TOR1AIP1*-associated nuclear envelopathies but also to cancer.

## Data Availability

The data that support the findings of this study are included in the published article and its Supplementary Data files. The MS proteomics data generated at the CRG/UPF Proteomics Unit have been deposited to the ProteomeXchange consortium [86] via the PRIDE [77] partner repository, having the dataset identifier PXD056445.

## Supplementary Materials

The following supporting information can be downloaded at: https://www.mdpi.com/article/10.3390/ijms252413235/s1.
